# Renal and Urinary Affections

**Published:** 1886-01

**Authors:** 


RENAL AND URINARY AFFECTIONS. By W. Howship Dickinson, M. D. Cantab., F. R. C. P., Physician to, and Lecturer on Medicine at St. George's Hospital, Consulting Physician to the Hospital for Sick Children, Corresponding Member of the Academy of Medicine of New York, Miscellaneous affections of the kidneys and urine. New York: William Wood & Co. 1885.

The growing importance of this class of disorders renders this work a most acceptable offering at present, and with its numerous instructive illustrations, the medical public should hail with much satisfaction this August addition to Wood's Library.

